# Are Sensory-Motor Relationships Encoded *ad hoc* or by Default?: An ERP Study

**DOI:** 10.3389/fpsyg.2019.00966

**Published:** 2019-05-03

**Authors:** Yurena Morera, Maartje van der Meij, Manuel de Vega, Horacio A. Barber

**Affiliations:** ^1^Departamento de Psicología Cognitiva, Social y Organizacional, Facultad de Psicología, Universidad de La Laguna, San Cristóbal de La Laguna, Spain; ^2^Instituto Universitario de Neurociencia, Universidad de La Laguna, San Cristóbal de La Laguna, Spain; ^3^Instituto de Tecnologías Biomédicas, Universidad de La Laguna, San Cristóbal de La Laguna, Spain; ^4^Basque Center on Cognition, Brain and Language, San Sebastian, Spain

**Keywords:** semantics, *ad hoc* categories, affordances, functional relations, N400

## Abstract

In this event-related potentials study we tested whether sensory-motor relations between concrete words are encoded by default or only under explicit *ad hoc* instructions. In Exp. 1, participants were explicitly asked to encode sensory-motor relations (e.g., “do the following objects fit in a pencil-cup?”), while other possible semantic relations remained implicit. In Exp. 2, using the same materials other group of participants were explicitly asked to encode semantic relations (e.g., “are the following objects related to a pencil-cup?”), and the possible sensory-motor relations remained implicit. The N400 component was sensitive to semantic relations (e.g., “desk” related to “pencil-cup”) both under implicit (Exp. 1) and explicit instructions (Exp. 2). By contrast, most sensory-motor relations (e.g., “pea” fitting in “pencil-cup”) were encoded *ad hoc* under explicit instructions (Exp. 1). Interestingly some sensory-motor relations were also encoded implicitly, but only when they corresponded to “functional” actions associated with high-related objects (e.g., “eraser” fitting in “pencil-cup”) and occurring at a late time window (500–650 ms; Exp. 2), suggesting that this type of sensory-motor relations were encoding by default.

## Introduction

Conceptual *productivity* is an important feature of human cognition that allows us to construct *ad hoc* categories combining the existent knowledge in new ways (Barsalou, 1983, 1991, 2010; Wu and Barsalou, 2009). For example, if you are sitting in a restaurant and you want to hide yourself from someone entering, you may construct the *ad hoc* category of *things that cover the face completely*. This category allows you to construe a newspaper or the menu as something that affords covering your face, whereas a fork does not (Barsalou, 1999). Also, human categorization is *flexible*, namely, the meaning of a given concept changes as its background changes (Barsalou, 1999). Following the previous example, the conceptualization of *newspaper* varies as the context of the concept changes from *something to read* to *something to cover the face* (Barsalou, 1999; Chrysikou, 2006).

In this line, many studies have demonstrated that sensory-motor properties are selectively activated when words appear in specific contexts or under specific task demands. For instance, the word *piano* in the sentence “*The man lifted the piano*” may activate different sensory-motor properties in memory than the same word in the sentence “*The man tuned the piano*.” Thus, people recall better the former sentence when receiving the cue “something heavy,” whereas they recall better the latter with the cue “something with a nice sound” (Barclay et al., 1974). Since Barclay et al.’s (1974) pioneer study, much evidence has been accumulated on how manipulating the context in which a word is presented affects which conceptual properties will be activated. For example, Yee et al. (2012) showed that, after focusing participants’ attention on color using the Stroop task, reading the name of an object (e.g., “emerald”) primed other objects that shared the same color (e.g., “cucumber”). In a similar vein, a neuroimaging study by Hoenig et al. (2008) explored conceptual flexibility during the verification of visual or action-related properties denoted by names of natural or artifactual objects. Previous literature had shown that visual properties were dominant for natural objects (e.g., *round* – orange), and functional properties were dominant for artifactual objects (e.g., *to play* – guitar). However, Hoenig et al. (2008) found activity in the visual cortex when asking for the visual properties of artifactual objects, whereas they found activation in the motor cortex when asking for functional properties of natural objects, thus demonstrating that non-dominant conceptual properties are strongly activated when they are explicitly requested by the task. In summary, there is evidence that processing word meaning not only activates fixed pre-stored conceptual semantic relations, but also could activate context-dependent properties, depending on the particular requirements of the situation.

Sensory-motor simulation mechanisms seem to play a central role in the construction of *ad hoc* categories (Glenberg, 1997; Barsalou, 1999; Glenberg and Robertson, 2000; Wu and Barsalou, 2009). Thus, to build the novel concept “*things that cover the face completely*,” simulations of the *objects*’ affordances combined with the goal of *hiding the entire face* might take place. In the case of a *newspaper a* simulation consistent with the category’s requirements is produced, and the object would be included in the *ad hoc* category; however, the affordances of a *fork* could not be combined to simulate an action consistent with such requirements and the object cannot be in included in the category. From this point of view, forming a new concept is not just constrained by associative mechanisms, but also by the use of affordances derived from sensory-motor experiences and body constraints to meet certain goals (Glenberg, 1997; Barsalou, 1999; Glenberg and Robertson, 2000; Wu and Barsalou, 2009).

Semantic categories (e.g., *screwdriver* and *hammer* as members of the category *tools*) involve conceptual relations, pre-stored in semantic memory, and their membership is automatically computed according to associative mechanisms (Barsalou, 2010; Abdel-Rahman and Melinger, 2011). By contrast, given the fact that *ad hoc* categories are novel by definition, they are not pre-stored in semantic memory and their membership is supposed to be established online, mainly by producing sensory-motor simulations.

The theoretical distinction between *ad hoc* relations and semantic relations between words has recently been developed by the Language and Situated Simulation (LASS) theory (Barsalou et al., 2008). According to this proposal, conceptual processing is supported by two systems. First, the linguistic system is immediately engaged when a word is perceived, and automatically processes word associations, which are pre-stored in long-term memory. Second, the simulation system, which entails the activation of modal information stored in the perceptual and motor brain areas in order to construct context-dependent or situated conceptualization, comes into play when the associative strength of words is not enough to respond to the task demands. According to the LASS theory, *semantic categories* would be processed mandatorily by the linguistic system, independently of the task demands, whereas *ad hoc categories* would be processed optionally by the simulation system, under specific task demands.

In this study we will test under which conditions *sensory-motor* relations are encoded by looking at the modulation of the amplitude of the N400 component in a categorization task. The N400 is a negative-going ERP component peaking around 400 ms after the onset of any potentially meaningful stimulus. A wide range of studies using single word presentation has shown that N400 amplitudes increase with the processing demands of word recognition and meaning activation (Barber and Kutas, 2007). Furthermore, the N400 amplitude for words typically correlates with how well a word fits with a prior context; the better it fits, the smaller the N400 amplitude (Kutas and Federmeier, 2011). Thus, the N400 amplitude is reduced for the second of two successive words (occurring as a pair or in a running list) when they are semantically related (e.g., Bentin et al., 1985; Luka and Van Petten, 2014). Although the N400 priming effects are typically larger with tasks that explicitly encourage semantic analysis, smaller but still reliable effects have been found when the instructions call for non-semantic tasks, indicating that these relations are encoded by default (Küper and Heil, 2009). Therefore, the modulation of the N400 by semantic priming has been clearly established (Holcomb, 1988; Chwilla et al., 1995).

However, the extent to which the N400 is modulated by the sensory-motor properties of word meaning has received less attention. One interesting exception is the ERP study performed by Kellenbach et al. (2000). These authors presented pairs of words referring to shape-related objects (e.g., button-coin) in a lexical decision task, reporting priming effects, consisting of N400 attenuation, based on the similarity in shape between the prime and the target objects. Especially relevant in our context are those studies about the sensory-motor properties of objects, involving words as experimental stimuli. For instance, the ERP study by Koester and Schack (2016) tested whether performing specific types of grip interacts with conceptual information while performing a lexical decision task. The words referred either to small or large objects and the response buttons required either precision or power grip, which was congruent or incongruent with the grasping action associated with the objects’ size. They found and early congruence effect (100–200 ms after noun onset) between the referred object size and the grip type; additionally, the same authors found a reduced N400 waveform, elicited by nouns for smaller compared with nouns for larger objects. On their side, Amsel et al. (2013), used a Go-NoGo paradigm dependent on a living/non-living judgment (semantic-related knowledge) or a graspable/ungraspable judgment (action-related knowledge). They found semantic-related information effects as early as 160 ms after the word onset, whereas action-related information was not available before 300 ms. All these results suggest that the N400 component is sensitive to the activation/integration of sensory-motor properties induced by specific goals or task demands even when object-related words are employed (see also: Aravena et al., 2010; Barber et al., 2010; Santana and de Vega, 2013).

In this ERP study we wanted to explore under what circumstances sensory-motor relations among objects, mediated by language, are processed by default or require *ad hoc* categorization processes. To this aim, we selected sets of target words which kept certain relations with a reference word (e.g., a pencil-cup). Specifically, two types of relations were manipulated orthogonally for each set: semantic relations and sensory-motor relations. The semantic relations consisted of sharing categorial membership or being associated with the same scenario as the reference object. For instance, “eraser” or “desk” are semantically related to the reference “pencil-cup” whereas “cake” or “comb” are not. Instead, the sensory-motor relations consisted of how well the target objects fit in the reference object; for instance, “eraser” or “battery” fit in a “pencil-cup” whereas “folder” or “cage” do not. We also selected another type of sensory-motor relation consisting of the capability of the reference object to produce physical changes in the target objects; for instance, “potato” or “lipstick” can be cut with a “knife,” whereas “helmet” or “wall” cannot. Crucially, we also manipulated the instructions to the participants asking them to encode either the sensory-motor relations or the semantic relations in two experiments, which used exactly the same sets of target words and the same reference object names. Previous literature has demonstrated that instructions and task contexts could determine the processing routes taken even by subliminal information (Kunde et al., 2003). Participants in Experiment 1 were given explicit instructions to encode a sensory-motor relation among several target objects and a reference object, for example, “do the following objects fit in a pencil-cup?” or “can the following objects be cut with a knife”? By contrast, in Experiment 2, participants were given explicit instructions to encode only semantic relations, such as “are the following objects related to a pencil-cup?” while the possible sensory-motor relations remained implicit. It should be noted that we asked participants to judge relational properties between objects, rather than characteristics of individual objects. On the other hand, concerning the sensory-motor relations, we did not ask participants to judge the similarity in form or function between the targets and the reference object (e.g., the one that exists between a dagger and a knife), but to evaluate whether the affordances of the targets and the reference object can be combined physically to accomplish certain goal (fitting in the reference object or being cut by it).

In Exp. 1, we expect that the explicit instructions to encode sensory-motor relations will prime words that fit the requested relation with the referent object (“eraser” or “pea” fitting in a “pencil-cup”), reducing the N400 in comparison with words without such a relation (“desk” or “mast”). According to the literature reviewed above, we could also expect implicit priming – and the corresponding N400 reduction – for semantically related (“eraser” or “desk” as related to “pencil-cup”) as compared to semantically unrelated words (“pea” or “mast”). It is also possible to get an interaction between both variables, for instance, if the “sensory-motor related – semantically related” condition (“eraser”) elicits the lowest N400 (because coherence is maximal), while the “sensory-motor unrelated – semantically related” condition (“desk”) elicits the highest N400 (because of conflicting information). In Exp. 2, we expect that the explicit semantic encoding instructions will strongly prime the semantically related words (“eraser” or “desk”), reducing N400 in comparison with semantically unrelated words (“pea” or “mast”). Concerning the implicit effect of the sensory-motor manipulation in Exp. 2, there are several possible results. First, a lack of N400 differences between sensory-motor related (“eraser” or “pea”) and unrelated words (“desk” or “mast”), as predicted by the LASS theory, would confirm that sensory-motor information is only activated *ad hoc*, and requires explicit instructions (as in Exp. 1). Second, the presence of N400 differences between sensory-motor related and unrelated words would indicate the spontaneous encoding of sensory-motor information, ruling out the *ad hoc* character of sensory-motor properties and suggesting that such properties are pre-stored in semantic memory and activated in a similar way as semantic relations. Third, interactive effects are also possible in Exp. 2; for instance, some sensory-motor relations could be implicitly encoded when they correspond to high-related objects, which could be stored in the semantic network along with semantic relations. Thus, “eraser” as something to be put into a “pencil-cup” could be encoded as an implicit sensory-motor related relation, because it corresponds to a common action in the participants’ repertoire. By contrast, low-related objects, such as putting a “pea” into a “pencil-cup” would not be encoded implicitly because this action is rarely performed and therefore it is not stored in the semantic network.

## Experiment 1: Explicit Sensory-Motor Encoding (Implicit Semantic Relations)

### Methods

#### Participants

Twenty-nine students from the University of La Laguna (mean age = 22 years; *SD* = 3.5; 20 women) were recruited from introductory psychology classes and earned extra credits for participation. The data from two participants were excluded from the ERP analyses because of excessive EEG artifacts. All participants were native Spanish speakers, had normal or corrected-to-normal eyesight, were neurologically healthy, and were right-handed, as determined by a Spanish translation of the Edinburgh Handedness Inventory (Oldfield, 1971; LQ score >50, *M* = 72.34; *SD* = 17.90).

The study was approved by the Human Research and Animal Welfare Ethics Committee (CEIBA) at University of La Laguna and carried out in accordance with the ethical standards of the Declaration of Helsinki. All participants gave written and informed consent to participate.

#### Stimuli

A total of 160 concrete nouns referring to inanimate objects were used in the study. They were divided into four 40-word independent sets corresponding to the following sensory-motor criteria of classification: (a) things which fit in a *pencil-cup*; (b) things which fit in a *sink*; (c) things which can be cut with a *knife*; and (d) things which can be passed through a *sieve* (see Appendix A, in Supplementary Material). Within each selected set, words were manipulated orthogonally according to their sensory-motor and semantic relations with a referent object (e.g., the pencil-cup). For instance, half of the words in a set were related to a sensory-motor criterion (they fit in a pencil-cup) and half were unrelated (they do not fit in a pencil-cup), and from each subset half were semantically related and half were semantically unrelated to the pencil-cup. In this way, we created four experimental conditions: *sensory-motor related* and *semantically related* words (SMT-R/SEM-R) (e.g., eraser), *sensory-motor related* and *semantically unrelated* words (SMT-R/SEM-U) (e.g., pea), *sensory-motor unrelated* and *semantically related* words (SMT-U/SEM-R) (e.g., desk) and *sensory-motor unrelated* and *semantically unrelated* words (SMT-U/SEM-U) (e.g., mast).

The sensory-motor and semantic relations with the corresponding referent objects were previously assessed in separate normative studies. Fifteen undergraduate psychology students, none of whom participated in the main experiments, rated words on a five-point scale for their sensory-motor relation with a given referent object (e.g., “rate the following objects according to whether they fit in a pencil-cup or not”), and another 15 students rated the same words for their semantic relation with a given referent object (e.g., “rate the following objects according to what extent they are related to a pencil-cup”). Targets rated higher than 3 were selected for the sensory-motor related condition (*M* = 4.9; *SD* = 0.07); targets rated lower than 0.25 were selected for the sensory-motor unrelated condition (*M* = 0.04; *SD* = 0.03); targets rated higher than 2.75 were selected for the semantically related condition (*M* = 3.12; *SD* = 0.34); and targets rated lower than 0.25 were selected for the semantically unrelated condition (*M* = 0.11; *SD* = 0.09). As expected, sensory-motor related and sensory-motor unrelated scores significantly differed [*t*(159) = 69.21, *p* < 0.0001], as well as did semantically related and semantically unrelated scores [*t*(159) = 21.15, *p* < 0.0001]. Words were matched on lexical frequency, length, number of neighbors, imageability, and concreteness according to the EsPal database (Duchon et al., 2013). Values of the four experimental conditions in each of these lexical variables were submitted to one-way ANOVAs and no statistically significant differences were identified (Appendix B, in Supplementary Material, shows the mean ratings for all these variables of each experimental condition).

#### Procedure

**FIGURE 1:**
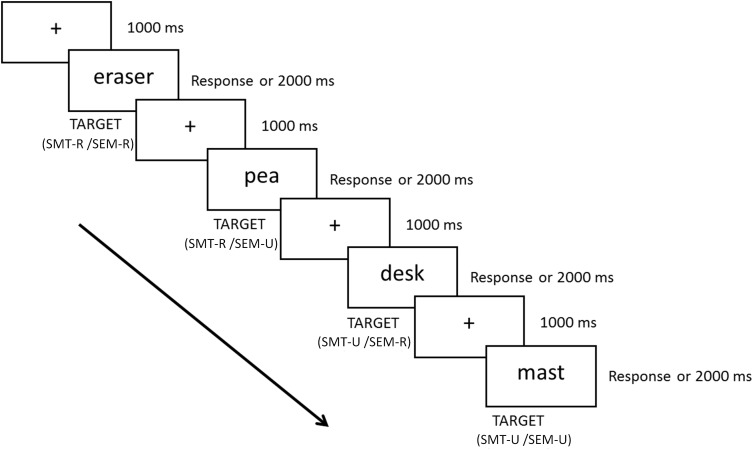
Schematic representation of a sequence of four trials, corresponding to a block of trials with instructions to judge whether the objects denoted by the words fit or not into a *pencil-cup*. The illustrated sequence includes a SMT-R/SEM-R (eraser), a SMT-U/SEM-U (pea), a SMT-U/SEM-R (desk) and a SMR-U/SEM-U (mast) trial.

The presentation of the stimuli and the recording of the responses were carried out using *Presentation* software^1^. All stimuli were presented on a high-resolution CRT monitor placed 80 cm in front of the participant at his/her eye level. Each experimental 40-word set started with the instruction to explicitly focus on a given referent object (e.g., “objects which fit in a *pencil-cup*”). After that, participants received a set of target words, which they had to judge as being either fitting or not to the referent object. The sequence of events in each trial is shown in Figure 1. Participants were asked to respond, as quickly and accurately as possible, by pressing the assigned “Yes” or “No” buttons with their left/right (or right/left for half of the participants) index fingers. Participants were asked to avoid eye movements and blinks while the fixation asterisk was not present. The four sets were presented in a different random order for each subject, as were the words within each set, that is: (a) things which fit in a *pencil-cup*; (b) things which fit in a *sink*; (c) things which you can cut with a *knife*; and (d) things which you can pass through a *sieve*). The experiment started with a short practice session (10 trials) and lasted approximately 30 min. Finally, after the participants had finished the experiment, they completed a posttest question on what they thought the experiment was about. None of the participants in this and in the next experiment declared to be aware of the experiment’s purpose.

#### EEG Recording and ERP Analyses

The electroencephalogram (EEG) was recorded with 27 Ag/AgCl electrodes embedded in an elastic cap (Easycap^2^) referenced to the left mastoid. Two pairs of electrodes above and below the left eye and on the outer canthi of each eye registered vertical and horizontal eye movements (EOG). The signal was amplified (BrainAmp amplifiers) and digitized at a sampling rate of 500 Hz, with a 0.01–100 Hz band pass filter. Impedance values were kept equal to or less than 5 kΩ at all electrode sites except for the four eye channels, which were kept below 10 kΩ. EEG data were stored and ERPs were later analyzed using Brain-Vision Analyzer 2.0 software^3^. The data were re-referenced offline to the average of the left and right mastoids, and passed through low cut-off (0.1 Hz, slope: 24 dB/oct) and high cut-off (30 Hz, slope: 24 dB/oct) filters. Artifacts were removed semi-automatically, with rejection values adjusted for each participant. This resulted in the exclusion of approximately 2.45% of the trials, which were evenly distributed across experimental conditions (minimum number of trials/condition = 30). The EEG data were segmented relative to reference marker positions in 900 ms time windows, corresponding with 100 ms before and 800 ms after the presentation onset of the target words. Baseline correction was performed using the average EEG activity in the 100 ms preceding target onset.

**FIGURE 2:**
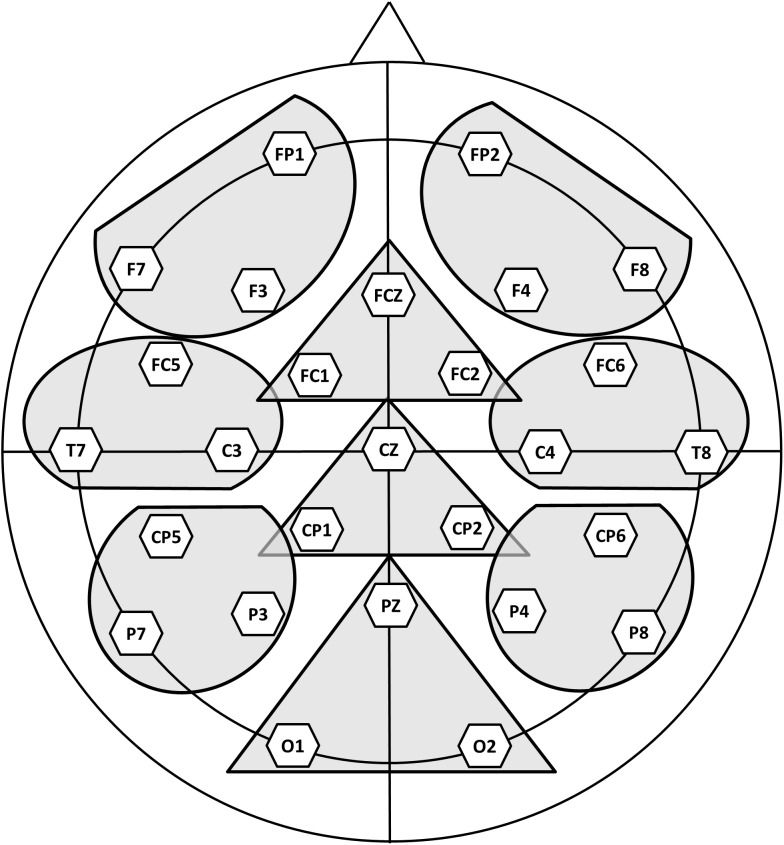
Regions of interest submitted to statistical analysis in Experiment 1 and Experiment 2 (see section “Experimental Procedure” for further details).

Separate ERPs were formed for each of the experimental conditions, each of the participants and each of the electrode sites. The main goal of these experiments was to study correlates of semantic processing associated to the experimental manipulations, therefore the modulation of the well-characterized N400 component was *a priori* the focus of attention for our analyses. However, the visual inspection of the grand averages revealed that, differences associated to some of the manipulations extend beyond the classical N400 time window. Point by point *t*-tests (see Appendix C, in Supplementary Material) showed that while some of the effects are present in a long time window others are restricted to a shorter and more standard time window (around 350 and 500 ms). Therefore, we choose to analyze two separate time windows, one between 350 and 500 ms and another between 500 and 650. These mean amplitude values (in microvolts) were subjected to separate analysis for each time window. ANOVAs for repeated measures were performed with the factors Sensory-motor relation (related, unrelated), Semantic relation (related, unrelated) and the topographical Region. For this topographical factor, nine different regions of interest were selected (see Figure 2), each comprising the mean of three electrodes: left anterior (Fp1, F7, F3), left central (FC5, T7, C3), left posterior (CP5, P7, P3), right anterior (Fp2, F8, F4), right central (FC6, T8, C4), right posterior (CP6, P8, P4), medial anterior (Fz, FC1, FC2), medial central (Cz, CP1, CP2), and medial posterior (Pz, O1, O2). When the sphericity assumption was violated, we report the Greenhouse-Geisser epsilon (*𝜀*) values to correct for the degrees of freedom. Since many *post-hoc* contrasts were carried out, we report the Hochberg *p*-values. We choose the correction proposed by Hochberg (1988) because it appropriately corrects for Type I errors, but does not generate a high Type II error rate. To complement the topographical descriptions of the effects, Appendix D (see Supplementary Material) shows additional analyses in which data of the individual electrodes were introduced in an ANOVA that included topographical factors.

### Results

#### Behavioral Data

**FIGURE 3:**
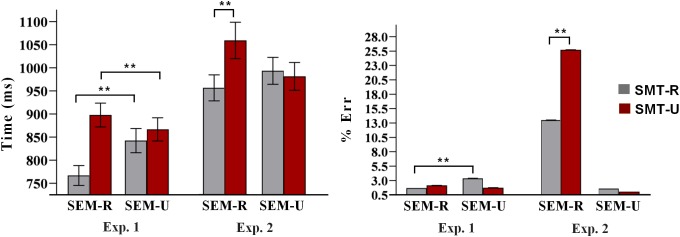
Mean latencies and errors percentage as a function of sensory-motor relation and semantic relation in Experiments 1 and 2. The vertical segments on the bars represent typical error. A double asterisk (^∗∗^) denotes significant effects at *p* < 0.0001. SMT-R: sensory-motor related; SMT-U: sensory-motor unrelated; SEM-R: semantic related; SEM-U: semantic unrelated.

Incorrect responses and response times (RTs) two standard deviations above or below the participants’ mean were excluded as outliers (7% of trials). Sensory-motor relation × Semantic relation ANOVAs were conducted on the RTs and error rates. The mean latencies and standard deviations are displayed in Table 1. Figure 3 depicts the mean latencies and errors percentage, for the four experimental conditions.

**Table 1:** Mean latencies and standard deviations in milliseconds, and error percentage as a function of Sensory-motor relation and Semantic relation, in Experiment 1 and Experiment 2 (SMT-R = Sensory-Motor Related; SMT-U = Sensory-Motor Unrelated).

	Semantic Related		Semantic Unrelated	
Experiment 1	*Mean (SD)*	Error %	*Mean* (*SD*)	Error %
SMT-R	767 (115)	1.7	843 (115)	3.4
SMT-U	899 (139)	2.2	868 (136)	1.8
**Experiment 2**
SMT-R	956 (129)	13.5	993 (134)	1.6
SMT-U	1060 (182)	25.7	982 (138)	1.0


The ANOVA on the RTs revealed a strong main effect of the Sensory-motor relation [*F*(1, 28) = 61.252, *p* < 0.0001]: responses were faster for related (*M* = 805 ms) than for unrelated targets (*M* = 884 ms). The main effect of Semantic relation was also significant [*F*(1, 28) = 20.38, *p* < 0.0001]: semantically related targets were responded to faster (833 ms) than semantically unrelated targets (856 ms). Finally, the interaction of the two factors was significant [*F*(1, 28) = 52.216, *p* < 0.0001]. Pair-wise comparisons revealed that sensory-motor related targets elicited faster reaction times when they were semantically related (*M* = 767 ms) than when they were semantically unrelated (*M* = 843 ms) [*t*(28) = 7.447, *p* < 0.0001]; however, sensory-motor unrelated targets were rejected faster when they were semantically unrelated (*M* = 868 ms) compared to when they were semantically related (*M* = 899 ms) [*t*(28) = 4.082, *p* < 0.0001].

The ANOVA on the error data revealed a significant Sensory-motor relation × Semantic relation interaction [*F*(1, 28) = 5.399, *p* < 0.028]. Pair-wise comparisons using the Wilcoxon test showed that the participants were more accurate in the sensory-motor related/semantically related condition (eraser/pencil-cup) (*M* = 1.7%) than in the sensory-motor related/semantically unrelated condition (pea/pencil-cup) (*M* = 3.4%) [*Z*(28) = 2.970, *p* < 0.003], while the comparison within the sensory-motor unrelated conditions did not approach significance [*Z*(28) = 0.749, *p* < 0.454].

#### ERP Data

**FIGURE 4:**
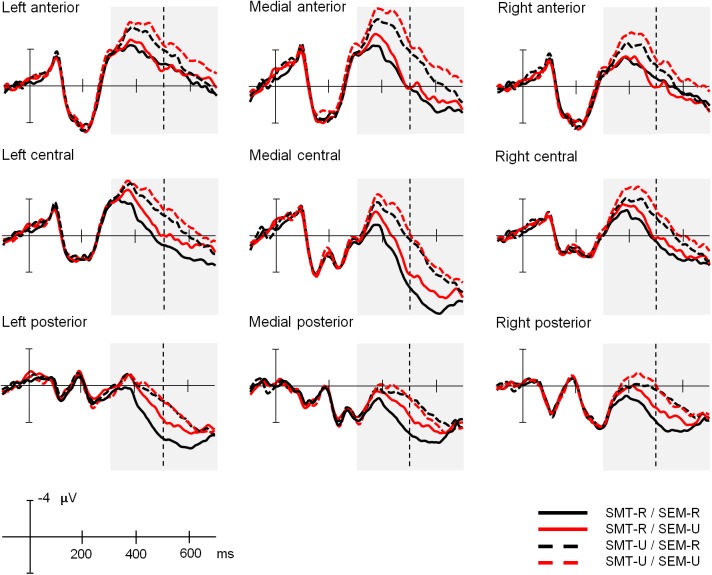
Experiment 1: Explicit sensory-motor encoding and implicit semantic relations. Grand average ERP waveforms for the four experimental conditions at the nine electrode areas analyzed. In this and following figures, negative polarity is plotted upward. SMT-R: sensory-motor related; SMT-U: sensory-motor unrelated; SEM-R: semantic related; SEM-U: semantic unrelated.

**FIGURE 5:**
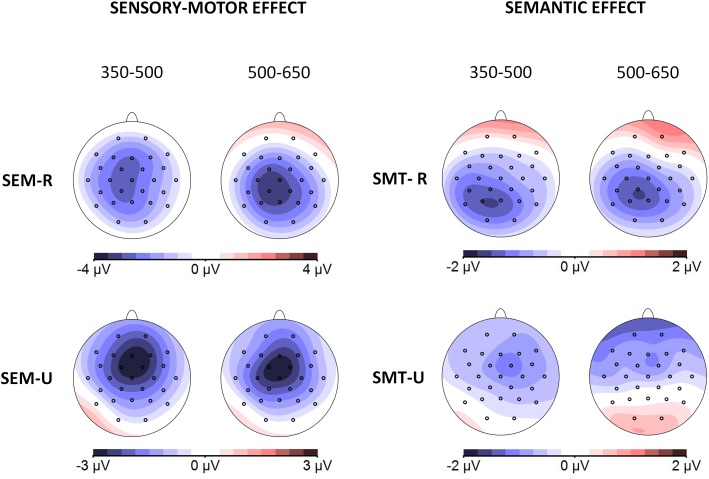
Scalp distributions of the different effects of Experiment 1 in the two analyzed time windows (350–500 and 500–650 ms). Topographical maps depict difference waves obtained from subtraction among homologous conditions: Sensory-motor effect (obtained by subtraction of sensory-motor unrelated from sensory-motor related conditions, left column) and Semantic effect (obtained by subtraction of semantic unrelated from semantic related conditions, right column) separately for related (top) and unrelated conditions (bottom).

Figure 4 depicts the grand averages time-locked to the onset of the target words for the four experimental conditions and the nine electrode regions of interest analyzed (here groups of electrodes have been averaged as they were entered in the analyses, but grand averages for all the individual electrode sites can be found in Appendix E of the Supplementary Material). Figure 5 shows the scalp distribution of the effects in the two analyzed time windows. The waveform between 350 and 500 ms after the word presentation captures the peak of a maximal negative deflection that can be identified as an N400 component. This N400 component was more enhanced in the sensory-motor unrelated targets than in the sensory-motor related targets. Moreover, ERP waves associated with semantically unrelated targets showed more negative amplitudes than those associated with semantically related targets. The differences in waveform among conditions extended to the later time window running from 500 to 650 ms. Both the sensory-motor and the semantic relation effects showed a widespread scalp distribution, and differences were greatest at central regions. Statistical analyses confirmed these observations.

##### 350–500 ms time window

The ANOVA yielded a main effect of the Sensory-motor relation [*F*(1, 26) = 56.37, *p* < 0.001], and a main effect of the Semantic relation [*F*(1, 26) = 10.05, *p* < 0.05]. Additionally, there was a two-way Sensory-motor relation × Region interaction [*F*(8, 208) = 7.46, *p* < 0.001], and a three-way Sensory-motor relation × Semantic relation × Region interaction [*F*(8, 208) = 4.96, *p* < 0.001]. *Post hoc* tests showed that whereas the sensory-motor relation effect was statistically significant for all the regions, the semantic relation effect was significant in all regions except for the left-anterior and the right-anterior regions.

##### 500–650 ms time window

The same ANOVA in this time window showed a main effect of the Sensory-motor relation [*F*(1, 26) = 40.9, *p* < 0.001], and a main effect of the Semantic relation [*F*(1, 26) = 6.41, *p* < 0.05]. There were also two-way interactions: Sensory-motor × Region [*F*(8, 208) = 5.7, *p* < 0.001] and Semantic relation × Region [*F*(8, 208) = 2.11, *p* < 0.05], as well as a three-way Sensory-motor relation × Semantic relation × Region interaction [*F*(8, 208) = 9.05, *p* < 0.001]. *Post hoc* tests showed that the sensory-motor relation effect was statistically significant for all regions, whereas the semantic relation effect was significant for the three left regions, and the medial-anterior and the medial-central areas.

### Discussion

Both the semantic relation and the sensory-motor relations induced broad modulations in the ERP waveform. Attending to the functional nature of our manipulations, the time course, morphology and the scalp distribution of these modulations we can identify the N400 component in the 350–500 ms time-window, indexing semantic processes. The neural processes reflected in the N400 activity might be cumulative and interactive processes, where incoming cues activate information previously stored in the long-term memory and update the ongoing mental representations and expectancies. As expected, there was a N400 effect associated with the sensory-motor relation under the explicit task instructions. Also worth noting is the N400 effect associated with semantic relations, which indicates an implicit encoding of these relations, since the task instructions did not explicitly focus on them. A clear relation between performance and neurophysiological measures was obtained. Both behavioral and ERPs measures were sensitive not only to the sensory-motor (explicit) but also to the semantic (implicit) manipulation, confirming the impact of our experimental manipulations.

Voltage differences in the same direction were also found at later latencies, between 500 and 650 ms, both for sensory-motor and semantic manipulations. Whereas sensory-motor effects showed the same scalp distribution in the two analyzed time windows, there were differences in relation to the semantic manipulation. *Post hoc* tests showed that semantic effects have a wide spread distribution (non-significant only at the left and right anterior areas) in the N400 time-window. However, in the 500–600 time-window the *post hoc* tests of semantic manipulation show a more left lateralized distribution both at anterior and posterior areas. These differences could mean that semantic effects in the two time windows are related to different brain sources and therefore to different cognitive processes. We will come back to this unresolved question in the discussion of Experiment 2.

Finally, the interaction between sensory-motor and semantic relations found both in behavioral measures and in ERP markers suggests that these two types of knowledge are related, at least under explicit instructions to code sensory-motor relations, suggesting that they could share neural networks. However, the experiment is not conclusive about whether sensory-motor relations are encoded *ad hoc* (that is, only induced by the explicit instructions) or whether this encoding occurs by default, as it was the case for the semantic relations. The next experiment tries to elucidate this question.

## Experiment 2: Implicit Sensory-Motor Relations (Explicit Semantic Encoding)

This experiment was a replication of the previous one, with an important novelty: participants were asked to categorize the target words in accordance with a general semantic relation criterion (e.g., “are the following objects related to a pencil-cup?”) rather than sensory-motor relation criteria.

We expect robust semantic relation effects on the ERP waveform, but the most important issue is to test whether the ERPs are also sensitive to the implicit sensory-motor properties. If, as the LASS theory predicts, sensory-motor relation effects are reduced or absent, this would mean that sensory-motor relations are not processed implicitly, but they are only encoded under *ad hoc* task demands. By contrast, the presence of sensory-motor relation effects would suggest that sensory-motor relations are processed implicitly online and likely are pre-stored in memory.

### Methods

#### Participants

Twenty-three students from the University of La Laguna (mean age = 19.4 years; *SD* = 3.3; 18 women) took part in the experiment. The data from one participant were removed from the ERP analyses due to excessive EEG artifacts. All participants were native Spanish speakers, had normal or corrected-to-normal eyesight, were neurologically healthy, and were right-handed, as determined by a Spanish translation of the Edinburgh Handedness Inventory (Oldfield, 1971; LQ score >50, *M* = 76.12; *SD* = 17.40).

The study was approved by the Human Research and Animal Welfare Ethics Committee (CEIBA) at University of La Laguna and carried out in accordance with the ethical standards of the Declaration of Helsinki. All participants gave written and informed consent to participate.

#### Stimuli and Procedure

The stimuli and the procedure were exactly the same as in Experiment 1, except that in each of the four sets of trials, participants were asked to make semantic relation judgments with respect to the same referent objects as in Experiment 1 (a *pencil-cup*, a *sink*, a *knife, or* a *sieve*). For instance, in a given block they were asked: “are the following objects related to a pencil-cup?”

### Results

#### Behavioral Data

The mean latencies and standard deviations for the four experimental conditions are displayed in Table 1 and depicted in Figure 3. Correct response latencies were trimmed as in Experiment 1. The ANOVAs on RTs revealed a significant main effect of Sensory-motor relation [*F*(1, 22) = 11.085, *p* < 0.003]: Participants responded faster to SMT-R (*M* = 974 ms) than to SMT-U targets (*M* = 1021 ms); the main effect of Semantic relation was not significant [*F*(1, 22) = 1.011, *p* = 0.327]. The important Sensory-motor relation × Semantic relation interaction was also significant [*F*(1, 22) = 12.168, *p* < 0.002]. Pair-wise comparisons revealed that for SEM-R trials, SMT-R words were responded to faster (956 ms) than SMT-U words (1060 ms) [*t*(22) = 3.805, *p* < 0.001]. However, for SEM-U trials the contrast between SMT-R and SMT-U words did not approach significance (*t* < 1).

The ANOVA on the error data revealed a significant main effect of Sensory-motor relation [*F*(1, 22) = 14.622, *p* < 0.001]: Participants responded more accurately to SMT-R words (7.5%) than to SMT-U sensory-motor unrelated targets (13.3%). The main effect of Semantic relation was also significant [*F*(1, 22) = 81.089, *p* < 0.0001], with less accurate responses in the SEM-R condition (19.6%) than the SEM-U condition (1.3%). The important Sensory-motor relation × Semantic relation interaction was also significant [*F*(1, 22) = 21.683, *p* < 0.0001]. Pair-wise comparisons using the Wilcoxon test revealed that for SEM-R trials, SMT-R words were responded more accurately (13.5%) than SMT-U words (25.7%) [*Z*(21) = 3.148, *p* < 0.002]. By contrast, for SEM-U trials SMT-R and SMT-U did not approach significant effects [*Z*(21) = 1.051, *p* < 0.293].

#### ERP Data

**FIGURE 6:**
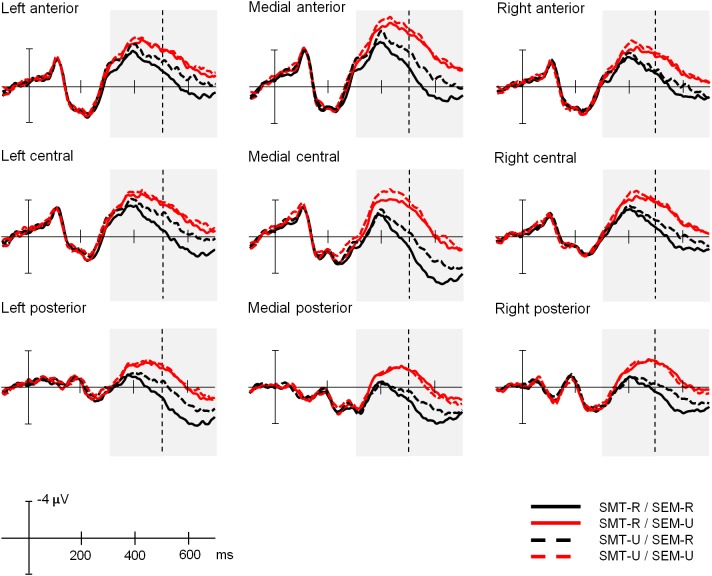
Experiment 2: Implicit sensory-motor relations and explicit semantic encoding. Grand average ERP waveforms for the four experimental conditions at the nine electrode areas analyzed. SMT-R: sensory-motor related; SMT-U: sensory-motor unrelated; SEM-R: semantic related; SEM-U: semantic unrelated.

**FIGURE 7:**
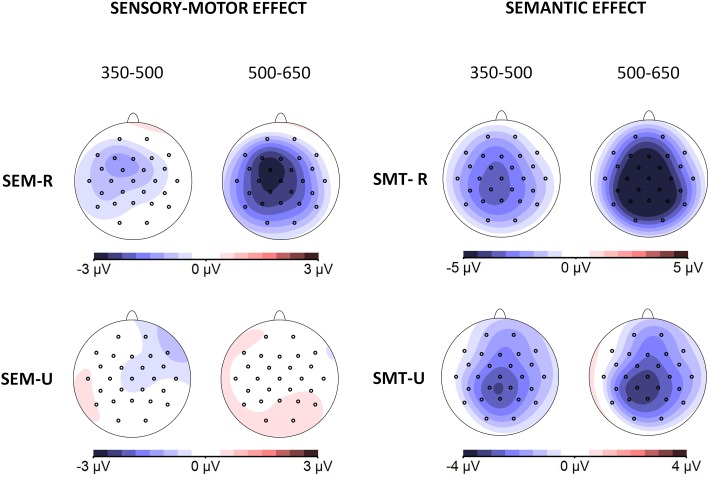
Experiment 2: Scalp distributions of the effects in the two analyzed time windows (350–500 and 500–650 ms). Topographical maps depict difference waves obtained from subtraction among homologous conditions: Sensory-motor effect (obtained by subtraction of sensory-motor unrelated from sensory-motor related conditions, left column) and Semantic effect (obtained by subtraction of semantic unrelated from semantic related conditions, right column) separately for related (top) and unrelated conditions (bottom).

Figure 6 depicts the grand averages time-locked to the onset of the target words for the four experimental conditions and the nine electrode regions of interest analyzed (grand averages for all the individual electrode sites can be found in Appendix E of the Supplementary Material). Figure 7 shows scalp distributions of the different comparisons in the two analyzed time windows. The amplitude of the N400 time window is larger for the semantically unrelated words than for the related ones, and this effect extends to the later time window analyzed. On the other hand, the sensory-motor relation effects seem to be modulated by semantic relations. In other words, sensory-motor high-related and low-related words do not differ in trials involving semantically unrelated words, while sensory-motor unrelated words elicit a more negative-going deflection than sensory-motor related words in trials with semantically related words. However, this effect occurs beyond the N400 time window, about 500 to 650 ms after the stimulus onset (see Figure 7). Statistical analyses confirmed these differences.

##### 350–500 ms time window

The ANOVA yielded a main effect of Semantic relation [*F*(1, 22) = 28.83, *p* < 0.001], but not of Sensory-motor relation [*F*(1, 22) = 2. 72, *p* = 0.11]. Additionally, there was a two-way interaction of Semantic relation × Region [*F*(8, 176) = 5.52, *p* < 0.001], although *post hoc* tests showed that the semantic effects were statistically significant in all the regions analyzed (see Appendix F, in Supplementary Material).

##### 500–650 ms time window

In this time window, there were significant main effects of Semantic relation [*F*(1, 22) = 66.8, *p* < 0.001] and of Sensory-motor relation [*F*(1, 22) = 9.18, *p* < 0.05]. These effects were qualified, however, by their interaction with Region: Semantic relation × Region [*F*(8, 176) = 7.1, *p* < 0.001]; and Sensory-motor relation × Region [*F*(8,176) = 2.52, *p* < 0.05]. *Post hoc* tests showed that the semantic relation effect was statistically significant in all the regions, and the sensory-motor relation effect was significant in the three left hemisphere regions, and at all the medial-anterior and all the medial-central regions. The Semantic relation × Sensory-motor relation interaction was also marginally significant [*F*(1, 22) = 3.41, *p* = 0.078], but given its theoretical relevance we also explored the corresponding simple effects. The pair-wise comparisons between sensory-motor related and sensory-motor unrelated words, when they were semantically related, yielded significant differences for all regions except the right-anterior area. By contrast, no difference was found between sensory-motor related and sensory-motor unrelated words when they were semantically unrelated. Namely, the effect of sensory-motor relation was constrained to semantically related words (see Figure 7, and Appendix F, in Supplementary Material).

### Discussion

As expected, a robust semantic relation effect was obtained in the N400 component of the ERPs, with less negative amplitudes associated with words that were semantically related to the referent object (e.g., “eraser” or “desk”) compared to those that were semantically unrelated (e.g., “pea” or “mast”). This effect extended to the later time window (500–650 ms), revealing that readers have more difficulties integrating semantically unrelated targets than semantically related targets. Concerning the sensory-motor relation variable, no statistically significant effects were observed in the N400 time window. However, at the later time window of 500–650 ms, there was a significant effect of sensory-motor relation (more negativity in sensory-motor unrelated words than in sensory-motor related words) constrained to semantically related trials. Moreover, the behavioral data analysis also obtained similar interactions: within the semantic related conditions, sensory-motor unrelated words (e.g., “desk”) produced larger response latencies and more errors than sensory-motor related words (e.g., “eraser”), suggesting that the cognitive system could implicitly encode sensory-motor properties of words, but only when they are semantically related. Thus, sensory-motor relations corresponding to high-related objects associated with “functional actions” (e.g., “eraser” fitting in a “pencil-cup”) were implicitly encoded in a delayed time window, whereas low-related objects, corresponding to novel or infrequent actions (e.g., “pea” fitting in a “pencil-cup”) were not encoded, confirming that they are only established *ad hoc* under explicit task demands.

Note that accuracy in the semantic judgments task (Exp. 2) was poorer than accuracy in sensory-motor judgments task (Exp. 1), as Table 1 and Figure 3 show. This can be due to the fact that semantic relations in our materials were quite heterogeneous, compared to the specificity of sensory-motor relations. An inspection of the materials in Appendix A (included in Supplementary Material), shows that in most cases semantic relations involved words frequently appearing in a given scenario; for instance, “desk” is related to the referent “pencil-cup” because both are present in offices. In other cases, semantic relations consisted of sharing membership with a superordinate category; for instance, “cutter” is related to the referent “knife” because both are cutting tools. Finally, in a few cases the semantic relation consisted of the association between a building and the referent object; for instance, “school” as related to the referent “pencil-cup.” The relative heterogeneity of semantic relations was statistically confirmed by the fact that sensory-motor related words have higher scores (*M* = 4.91) than semantically related words (*M* = 3.12) in the normative study described in section “Stimuli” [*t*(159) = 18.37, *p* < 0.0001]. These data could also indicate that responses in Exp. 1 are based on a simple binary distinction (yes/no), while responses in Exp. 2 involve to categorize words along a continuum, according to whether or not they belong to a semantic category. This would explain why accuracy was higher in Exp. 1 than in Exp. 2. However, the materials were built in such a way as to ensure that semantic relations receive an appropriate experimental manipulation (see section “Stimuli” and Appendix B, in Supplementary Material). Moreover, we empirically know that the ERP signatures were sensitive to these diverse semantic relations even under implicit encoding conditions (Exp. 1). Also, using a generic semantic question has the advantage of keeping exactly the same experimental design and the same materials (targets and reference objects) as in the previous experiment, making them more comparable.

In contrast to the results of experiment 1, *post hoc* analyses did not show differences in the scalp distribution of the semantic effects across the two analyzed time windows. However, in this case, the sensory-motor effects (observed only in the second time window) show a more anterior and left lateralized distribution. Therefore, it could be argued that the effects of the implicit conditions in the second time window (semantic manipulation in experiment 1 and sensory-motor manipulation in experiment 2) have a more anterior and left lateralized distribution. Since the N400 may reflect the contribution of several brain sources, in our tasks these sources could contribute in a different way over time depending on whether the encoding is explicit or not. However, with the present data we cannot speculate on the exact functional nature of those potential different sources. Alternatively, these differences in scalp distributions could be related to the smaller size of the implicit effects and the dynamics of the electric fields on the scalp over time. In any case, we can understand the effects in the second time window as a continuation of the semantic processing initiated in the first window. Similar late effects related to semantic manipulations have been previously reported (e.g., De Sanctis et al., 2013; Koester and Schack, 2016).

## General Discussion

This study explored the brain’s response to sensory-motor relations between concrete words under explicit and implicit conditions in a categorization task. To this end, the same sets of words, differing orthogonally in both sensory-motor and semantic relations, were presented to the participants under two different task instructions, involving explicit sensory-motor judgments (Exp. 1) and explicit semantic judgments (Exp. 2). The main results were as follows. First, the brain’s response to semantic relations (enhanced N400 for semantically unrelated words extending at least until 650 ms after stimulus onset) occurred both under implicit (Exp. 1) and explicit semantic encoding instructions (Exp. 2) being corroborated by the behavioral results. Second, under the instructions involving explicit sensory-motor encoding (Exp. 1), there was a robust effect of sensory-motor relation starting in the N400 time window and extending beyond it. However, sensory-motor relations were not implicitly encoded (Exp. 2) in the N400 time window. Only when the words were semantically related, referring to high-related objects, sensory-motor relations were implicitly encoded but in a later time window (500–650 ms). Moreover, the behavioral results confirm a similar implicit sensory-motor effect (faster and more accurate responses to sensory-motor related words than to sensory-motor unrelated words) constrained to semantically related words. The most interesting results in this paper concern implicit relations, which are not driven by the particular task instructions, and allow us to explore which relations are encoded by default, and which ones requires *ad hoc* strategies.

The scalp distributions of the reported effects show a widespread distribution over the scalp, with maximum differences at central electrodes, which is consistent with previous studies on semantic priming (e.g., Bentin et al., 1985; Holcomb, 1988; Chwilla et al., 1995; Küper and Heil, 2009; Luka and Van Petten, 2014). Taken together both experiments, there is no evidence of that semantic and sensory-motor effects differ in their scalp distributions *per se* (see also Figure 8 at the Appendix G of the Supplementary Material), and therefore, we cannot propose qualitatively different functional mechanisms for these effects.

Our results could only be partially explained by the LASS theory (Barsalou et al., 2008). The early semantic relation effect obtained under both implicit and explicit instructions supports the automatic activation of the linguistic system, which immediately triggers associations between the target word and the referent object (e.g., eraser → pencil-cup or desk → pencil-cup), as predicted by the LASS theory (Barsalou et al., 2008; see also: Holcomb, 1988; Chwilla et al., 1995; Küper and Heil, 2009). Also, the presence of an early sensory-motor effect under explicit instructions is predicted by the LASS theory, given that the task demand calls for an *ad hoc* categorization that explicitly requires the use of the sensory-motor simulation system (e.g., a pea fitting in a pencil-cup is encoded) (Barsalou, 1999; Wu and Barsalou, 2009). This early effect faded when the task demands did not call explicitly for *ad hoc* categorization (Exp. 2), indicating that the sensory-motor simulation does not work implicitly (e.g., a pea related to a pencil-cup is not encoded), which also fits the theory. However, the LASS theory does not predict the delayed effect of sensory-motor relatedness under implicit instructions obtained for semantically related words; for instance, the difference obtained in the later ERPs time window between “eraser” (it fits a pencil-cup) and “desk” (it doesn’t fit a pencil-cup) even though both are semantically related targets. According to this theory, when the linguistic system can accomplish the task demands on its own, there may be no need to use the simulation system, whereas in the current case, even when the linguistic system successfully encoded the explicit semantic relations, there was a later encoding of sensory-motor properties, which was not required by the task. This encoding occurred in a later time window, indicating that after processing the semantic properties, participants processed the sensory-motor properties associated with those relations that also have a functional meaning. Let us consider some possible explanations of this delayed sensory-motor relation effect.

One possible explanation for the above results is that semantic memory stores not only “linguistic” semantic relations (“eraser” belongs to the same category as “pencil-cup”) but also stores, as world knowledge, some significant sensory-motor relations. This would be consistent with the classical study by Hagoort et al. (2004) who found N400 modulations associated with both semantic- and world-knowledge violations. Note that in the current study these sensory-motor properties are not individual features of an object (e.g., its size or color) but they are relational, involving potential interactions between objects. Namely, only action which are typically performed with high-related objects in everyday situations would be processed by default as world knowledge (e.g., putting erasers in pencil-cups). However, according to our results, the two kinds of relations are not equally accessible. Whereas semantic relations are implicitly encoded within a 400 ms time window (Exp. 1), sensory-motor relations between high-related objects are implicitly encoded at a later moment (Exp. 2), suggesting a time-consuming spread activation process (Collins and Loftus, 1975) from the semantic nodes to the sensory-motor property nodes.

The literature on the possible interaction between conceptual and sensory-motor information is controversial. Our results support the idea, defended by the LASS theory, that we first access to conceptual information and then to sensory-motor properties of the referred words. Similar results were obtained by Amsel et al. (2013) using a go/nogo task. They found earlier category-related information effects and delayed action-related information (about 160 and 300 ms. after the word onset, respectively). However, Koester and Schack (2016) found a very early interaction (100–200 ms. after noun onset) between motor-related information (size of referred objects) and type of grip response (congruency effect), but also reported a later N400 effect associated with objects size, which could be related to the integration of motor representations and conceptual information during the processing of objects functional properties. There are many differential features of the materials, experimental paradigms and task demands among the current study and those reported in the literature, which may contribute to modulate the ERP results differently. For instance, in Amsel et al. the go-nogo paradigm involves inhibition/control processes indexed by the N200, and in Koester and Schack (2016) the interaction occurs on motor response components of the ERP. By contrast, our task did not manipulate any parameter of response (always consisted of pressing one of two alternative keys), and as would be expected only late semantic components are modulated by the task demands. In spite of that, we reported in Experiment 2 a delayed processing of sensory-motor features (only for high-related objects) compared to the semantic features, with the novelty that this processing occurred implicitly.

Not surprisingly, related sensory-motor properties are not implicitly encoded when the word and the referent object do not have any semantic relation (putting a pea into a pencil cup). It does not make sense to encode or store in long-term memory all the potential sensory-motor interactions among objects when most of them are irrelevant for a particular situation. For instance, although “peas”, “lipsticks”, “coins”, and “shells” could be put into a “pencil-cup” it would not be useful to activate by default this sensory-motor relation, unless you are asked to do so “*ad hoc*”. However, it is quite reasonable that a few privileged sensory-motor relations are routinely encoded as functional properties of objects. For instance, small objects semantically related to scholar activity (e.g., eraser, pencils, pens, clips, etc.) are typically put inside pencil-cups, and these familiar containment-container relations could become strongly associated with the corresponding semantic nodes and encoded by default.

Another way to consider the processing of high related (or functional) and low related (novel) sensory-motor properties is in terms of affordances or potential motor acts associated with objects (Gibson, 1979; Glenberg, 1997; Glenberg and Robertson, 2000; Cisek and Kalaska, 2010). For instance, some objects like glasses, jars or phones are graspable, and they trigger activations of the hand motor neural networks. Some studies have demonstrated that both manipulable objects and pictures of manipulable objects placed in the peripersonal space automatically trigger their motor affordances (Buccino et al., 2009; Costantini et al., 2011). More interesting for this study, it has been found that even words referring to manipulable objects can trigger their motor and functional properties (Glover et al., 2004; Boronat et al., 2005; Anelli et al., 2010).

Notice, however, that the notion of affordance underlying the above studies consists of eliciting a simple grasping-related activity in the motor brain in the presence of an object or its verbal label. By contrast, the sensory-motor relations analyzed in the current study are rather more complex, and could be better described as assessing whether the combination of affordances of two objects allows one to “simulate” a given action based on the objects’ sensory-motor properties according to a given goal. For instance, a pencil-cup is a container that affords putting smaller-size objects inside, and an eraser or a pea (but not a mast) fits this requirement and affords grasping and being put inside; or a knife affords cutting soft solid objects, and a cheese or a sponge (but not a helmet or a scalpel) fits this requirement and affords grasping and being cut. This idea is akin to the concept of “functional affordance” proposed by Mizelle et al. (2013) to refer to our knowledge of functional actions associated with tools use. According to the authors, to evaluate a correct (e.g., screwdriver held by handle) or an incorrect (e.g., screwdriver held by bit rather than handle) use of a tool we need to process not only its physical characteristics, but also to know when and how to use it. Our results suggest that functional affordances could be triggered implicitly by words when the referred objects are semantically related, namely, functional relations, whereas the combination of affordances can also be computed *ad hoc* for semantically unrelated objects, namely, novel relations, under explicit encoding instructions.

## Conclusion

This study has shown that semantic relations are always encoded, modulating the N400 component of the ERPs, independently of the task demands, thus confirming previous findings in the literature. This encoding happens despite the fact that, in this study, the semantic relations were quite unspecific. By contrast, sensory-motor relations seem to have a dual nature. First, they are encoded in the same N400 time window, but only under explicit task demands, for both semantically related and semantically unrelated words, suggesting an *ad hoc* simulation process. Second, sensory-motor relations are also encoded under implicit task demands, but in a later time window and only when they refer to high related objects relations or functional properties that are strongly associated with the semantically related target words. This delayed encoding suggests spread activation processes from the semantic nodes to the functional nodes pre-stored in semantic memory, and it is also compatible with the activation and combination of affordances derived from the referred objects. These results allow for a qualification of the notions of *ad hoc* categories and sensory-motor simulations postulated by the LASS theory. In particular, they show that high related objects relations or functional sensory-motor properties denoted by words could be processed implicitly, after one has succeeded in computing their semantic relations.

The ERP methodology employed in this study provides an accurate temporal view on the ongoing semantic and sensory-motor encoding processes. However, it does not inform us on the specific underlying brain mechanisms. An important research avenue is to explore in detail the neurobiological basis underlying linguistic encoding of sensory-motor and semantic relations. Neuroimaging and non-invasive brain stimulation methods could be useful to reveal the role of motor, pre-motor and cognitive control network in these processes (Hauk et al., 2004). Especially relevant would be to investigate whether the motor and pre-motor brain areas are activated during the *ad hoc* encoding (explicit) of sensory-motor properties and the implicit encoding of functional properties of words. Although the current study was performed with young healthy participants, the results could have implications in clinic contexts. Particularly, patients with neurodegenerative diseases or those suffering brain stroke frequently exhibit conceptual or ideomotor apraxia, including impairment of object or action knowledge, difficulty to match objects and actions or to understand tools use (Gross and Grossman, 2008; Stamenova et al., 2012). Moreover, language plays a role in apraxia, given the fact that these patients are unable to follow verbal commands to perform gestures or manual tasks. Further research could explore whether apraxia patients show anomalous modulations of ERP while judging sensory-motor relations either high related or *ad hoc*.

## Ethics Statement

The study was approved by the Human Research and Animal Welfare Ethics Committee (CEIBA) at University of La Laguna, and carried out in accordance with the ethical standards of the Declaration of Helsinki. All participants gave written and informed consent to participate.

## Author Contributions

YM, MdV, and HB designed the research. YM and MvdM performed the research. MvdM and HB analyzed the data. YM, MdV, and HB wrote the manuscript.

## Conflict of Interest Statement

The authors declare that the research was conducted in the absence of any commercial or financial relationships that could be construed as a potential conflict of interest.

## Footnotes

1 http://www.neurobs.com

2 http://www.easycap.de

3 http://www.brainproducts.com
