# Supplementary material for: Are Sensory-Motor Relationships Encoded ad hoc or by Default?: An ERP Study
Source: Front Psychol. 2019 May 3;10:966. doi: 10.3389/fpsyg.2019.00966 (PMC6511810; doi:10.3389/fpsyg.2019.00966)

Appendix A

Target words used in Experiments 1 and 2 in the four experimental conditions: Sensory-Motor Related & Semantically Related (SMT-R / SEM-R); Sensory-Motor Related & Semantically Unrelated (SMT-R / SEM-U); Sensory-Motor Unrelated & Semantically Related (SMT-U / SEM-R) and Sensory-Motor Unrelated & Semantically Unrelated (SMT-U / SEM-U). They are in the original Spanish followed by the English translation between brackets.

Referent Object	SMT-R / SEM-R	SMT-R / SEM-U	SMT-U / SEM-R	SMT-R / SEM-U
(1) Pencil-cup	Afilador (sharpener)	Aguja (needle)	Academia (academy)	Cabina (booth)
	Bolígrafo (pen)	Anillo (ring)	Atril (bookrest)	Flotador (float)
	Cera (crayon)	Anzuelo (fishhook)	Aula (classroom)	Jaula (cage)
	Compás (compass tool)	Cuchara (spoon)	Carpeta (folder)	Mástil (mast)
	Goma (rubber)	Guisante (pea)	Escuela (school)	Pértiga (pole)
	Pincel (paintbrush)	Llavero (keychain)	Librería (library)	Sombrero (hat)
	Regla (rule)	Navaja (penknife)	Papelería (stationery)	Tarta (cake)
	Rotulador (marker)	Peine (comb)	Pizarra (board)	Telón (curtain)
	Sello (stamp)	Pila (battery)	Pupitre (desk)	Tobogán (toboggan)
	Tijeras (scissors)	Tornillo (screw)	Silla (chair)	Tronco (trunk)
(2) Sink	Bombilla (bulb)	Botón (button)	Alacena (cupboard)	Banco (bank)
	Botella (bottle)	Candado (padlock)	Aseo (bathroom)	Camilla (stretcher)
	Caldero (pot)	Cassette (cassette)	Bombona (gas bottle)	Canoa (canoe)
	Cenicero (ashtray)	Corbata (tie)	Cocina (kitchen)	Carreta (cart)
	Jarra (jug)	Dado (dice)	Despensa (pantry)	Casino (casino)
	Plato (plate)	Dardo (dart)	Horno (oven)	Circo (circus)
	Sopera (tureen)	Dedal (thimble)	Nevera (fridge)	Farola (streetlight)
	Tazón (bowl)	Ficha (token)	Repisa (shelf)	Palco (theater box)
	Tenedor (fork)	Gorra (cap)	Sofá (sofa)	Remo (oar)
	Vaso (glass)	Mechero (lighter)	Tele (TV)	Templo (temple)
(3) Knife	Cebolla (onion)	Billete (banknote)	Bisturí (scalpel)	Alcantarilla (sewer)
	Hueso (bone)	Carmín (lipstick)	cutter (cutter)	Ancla (anchor)
	Jamón (ham)	Cerilla (match)	Daga (dagger)	Cancha (court)
	Lechuga (lettuce)	Cigarro (cigarette)	Espada (sword)	Casco (helmet)
	Manzana (apple)	Corcho (cork)	Guillotina (guillotine)	Cueva (cave)
	Papas (poteto)	Esponja (sponge)	hacha (ax)	Misil (missile)
	Pepino (cucumber)	Hilo (thread)	Hoz (sickle)	Muro (wall)
	Pera (pear)	Naipe (card)	machete (machete)	Podio (podium)
	Queso (cheese)	Puro (cigar)	Sable (cutlass)	Tanque (tank)
	Tortilla (omelette)	Tiza (chalk)	Sierra (saw)	Yunque (anvil)
(4) Sieve	Batido (shake)	Aguarrás (turpentine)	Cazo (saucepn)	Agenda (agenda book)
	Caldo (broth)	Betadine (betadine)	Copa (goblet)	Arpa (harp)
	Compota (compote)	Champú (shampoo)	Cuenco (bowl)	Bastón (cane)
	Jugo (juice)	Colonia (cologne)	Embudo (funnel)	Broche (brooch)
	Leche (milk)	Diesel (diesel)	Escurridor (drainer)	Cepillo (brush)
	Manzanilla (chamomile)	Disolvente (solvent)	Filtro (filter)	Farol (lantern)
	Papilla (pap)	Gel (gel)	Losero (drainer)	Látigo (whip)
	Salsa (sauce)	Lejía (bleach)	Rallador (grater)	Manguera (hose)
	Sopa (soup)	Talco (talcum)	Taza (cup)	Martillo (hammer)
	Zumo (juice)	Tinta (ink)	Tetera (kettle)	Percha (hanger)

Appendix B

Mean ratings and standard deviations for Sensory-motor relation, Semantic relation, Written frequency, Length, Number of neighbors, Imageability, and Concreteness for targets used in Experiments 1 and 2 (SMT-R / SEM-R = Sensory-Motor Related & Semantically Related; SMT-R / SEM-U = Sensory-Motor Related & Semantically Unrelated; SMT-U / SEM-R = Sensory-Motor Unrelated & Semantically Related; SMT-U / SEM-U = Sensory-Motor Unrelated & Semantically Unrelated).

	Condition			
	SMT-R / SEM-R	SMT-R / SEM-U	SMT-U / SEM-R	SMT-U / SEM-U
	M (SD)	*M (SD)*	*M (SD)*	*M (SD)*
Sensory-motor relation	4.92 *(0.09)*	4.90 *(0.05)*	0.04 *(0.04)*	0.04 *(0.03)*
Semantic relation	3.27 *(0.21)*	0.10 *(0.07)*	2.98 *(0.48)*	0.12 *(0.12)*
Written frequency	0.83 *(0.42)*	0.78 *(0.46)*	0.77 *(0. 47)*	0.81 *(0.34)*
Length	6.03 *(1.59)*	6.08 *(1.46)*	6.08 *(1.68)*	5.95 *(1.41)*
Number of neighbors	3.82 *(4.26)*	3.17 *(4.10)*	3.40 *(4.54)*	3.12 *(2.93)*
Imageability	5.89 *(1.21)*	5.87 *(1.38)*	5.78 *(0.64)*	5.99 *(0.45)*
Concreteness	5.83 *(0.64)*	5.78 *(0.68)*	5.86 *(0.57)*	5.81 *(0.61)*

Appendix C

T-tests performed on each data point. Data were collected every 2 ms (p <.05).

Examples of the two main effects of Experiment 1 are presented.

1) Sensory-Motor effect: Sensory-motor related Vs. sensory-motor unrelated

Significant points are continually found between 350 and 650 ms in most of the electrode sites.

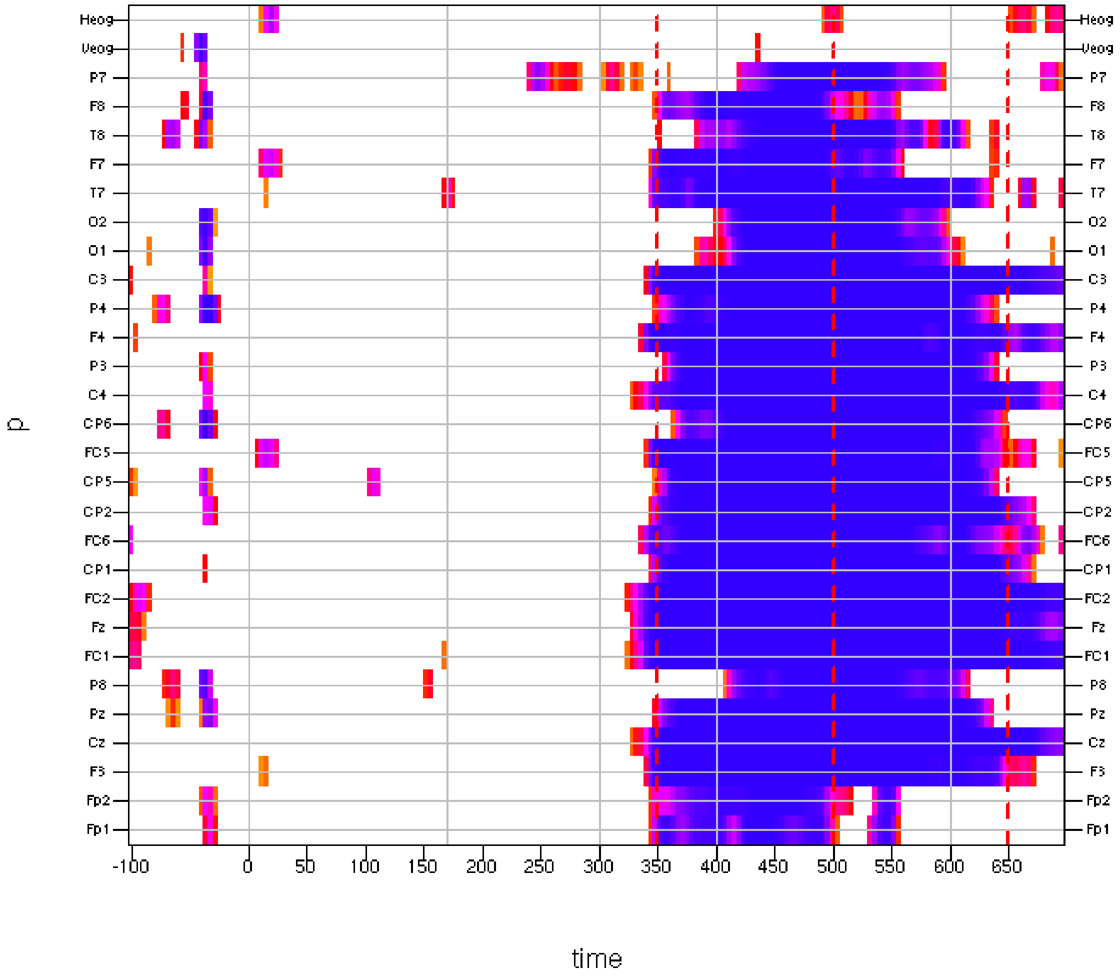

2) Semantic effect: Semantic related Vs. semantic unrelated

Significant points are continually found only in a shorter time window between 350 and 500 ms in several electrode sites.

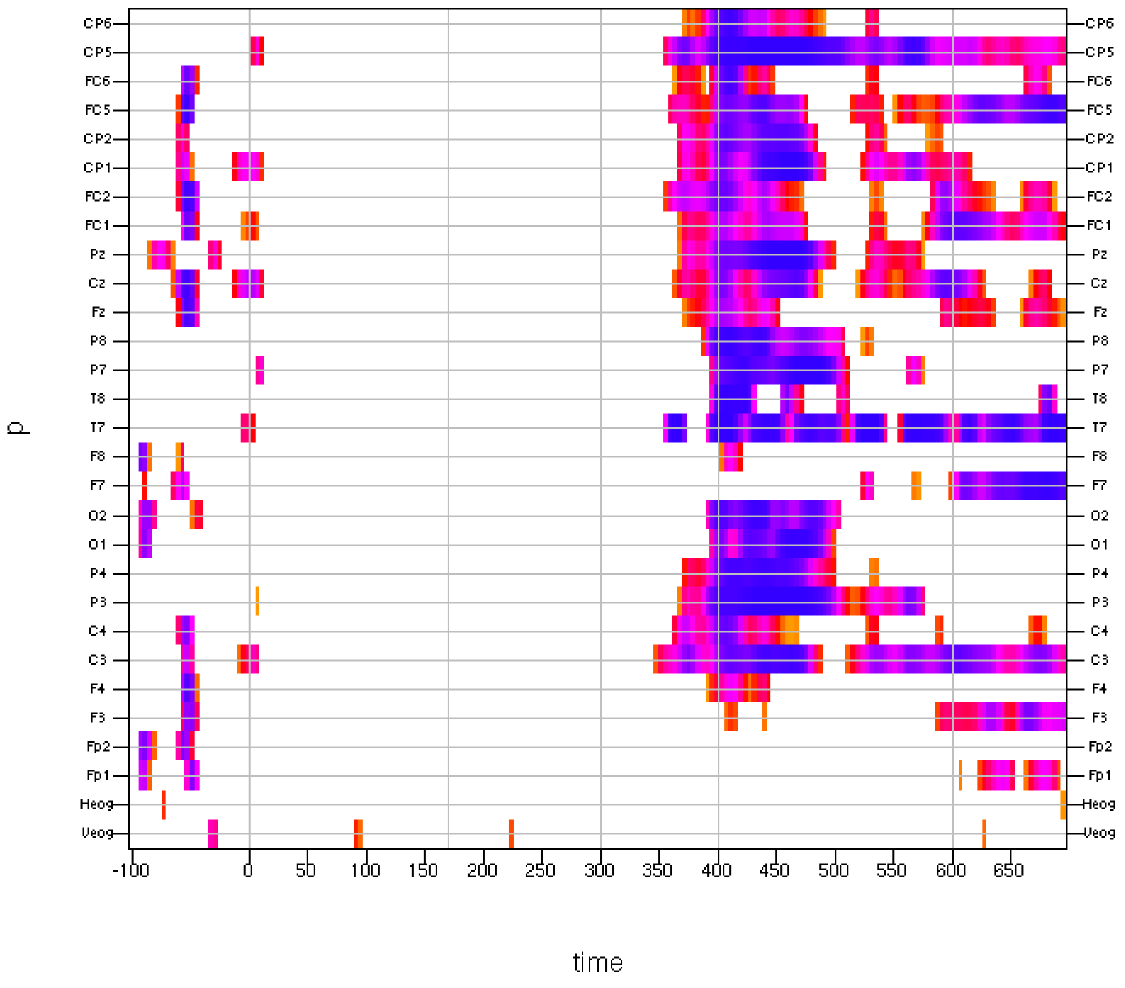

Appendix D

Alternative analysis with topographical factors for Experiment 1 and Experiment 2, in the two ERP temporal windows, with Sensory-Motor (related, unrelated) and Semantic relatedness (related, unrelated) as within-subject factors, and three topographical factors: Hemisphere (left, right), and Anterior Posterior (AP) (frontal, frontal-central, central, central-parietal, parietal) and Distance to midline (DM) (one position from midline, two positions from midline).

1) EXPERIMENT 1: Alternative analysis with topographical factors

Omnibus analysis of variance (ANOVA) with Sensory-Motor (related, unrelated) and Semantic relatedness (related, unrelated) as within-subject factors, and three topographical factors: Hemisphere (left, right), and Anterior Posterior (AP) (frontal, frontal-central, central, central-parietal, parietal) and Distance to midline (DM) (one position from midline, two positions from midline).

Results

Time window: 350-500

- Main effects: Sensory-Motor ($F(1,26)=60.39$, $p<.001$) and Semantic relatedness ($F(1,26)=11.23$, $p<.01$). Main effects averages are shown in the next Table:

Sensory-Motor related	-0.62
Sensory-Motor unrelated	-2.36
Semantic related	-1.12
Semantic unrelated	-1.85

- Double Interaction: Sensory-Motor and DML ($F(1,26)=50.54$, $p<.001$) and Sensory-Motor and AP ($F(4,104)=7.64$, $p<.01$; $\varepsilon=.33$), post hoc test showed for both interactions a significant effect ($p<.001$) in all levels.
- Triple interaction: Sensory-Motor and Semantic relatedness with Hemisphere ($F(1,26)=5.48$, $p<.05$) and Sensory-Motor and Semantic Relatedness with AP ($F(4,104)=4.51$, $p<.05$; $\varepsilon=.35$) post hoc test showed for this interaction a significant Sensory-Motor effect ($p<.001$) in all levels of the two topographical factors.
- Quadruple interaction: Sensory-Motor, AP, DML and Hemisphere ($F(4,104)=3.97$, $p<.01$; $\varepsilon=.78$). Not possible to perform post-hoc test.

Time window: 500-650

- Main effects: Sensory-Motor ($F(1,26)=46.19$, $p<.001$) and Semantic relatedness ($F(1,26)=7.23$, $p<.05$). Main effects averages are shown in the next Table:

Sensory-Motor related	1.97
Sensory-Motor unrelated	0.28
Semantic related	1.43
Semantic unrelated	0.81

- Double Interaction: Sensory-Motor and DML ($F(1,26)=53.89$, $p<.001$) and Sensory-Motor and AP ($F(4,104)=4.77$, $p<.05$; $\varepsilon=.31$). Post hoc test showed for these interactions a significant effect of Sensory-Motor ($p<.001$) in all levels of AP.
- Triple interaction: Sensory-Motor and Semantic relatedness with AP ($F(4,104)=9.10$, $p<.01$; $\varepsilon=.31$)**.** Results post hoc tests triple interaction for time window 550-600 are shown in the following Table:

AP	Post-hoc
Frontal	Semantic relatedness effect only in Sensory-Motor unrelated condition: $p<.05$<br>Sensory-Motor effect only in semantic unrelated condition: $p<.001$
Frontal-central	Semantic relatedness effect only in Sensory-Motor unrelated condition: $p<.05$<br>Sensory-Motor effect in semantic related and semantic unrelated conditions: $p<.001$
Central	Semantic relatedness effect only in Sensory-Motor unrelated condition: $p<.05$<br>Sensory-Motor effect in semantic related and semantic unrelated conditions: $p<.001$
Central-parietal	Semantic relatedness effect only in Sensory-Motor related condition: $p<.05$<br>Sensory-Motor effect in semantic related and semantic unrelated conditions: $p<.01$
Parietal	Semantic relatedness effect only in Sensory-Motor related condition: $p<0.01$<br>Sensory-Motor effect only in semantic related condition: $p<.001$

2) EXPERIMENT 2: Alternative analysis with topographical factors

Omnibus analysis of variance (ANOVA) with Sensory-Motor (related, unrelated) and Semantic relatedness (related, unrelated) as within-subject factors, and three topographical factors: Hemisphere (left, right), and Anterior Posterior (AP) (frontal, frontal-central, central, central-parietal, parietal) and Distance to midline (DM) (one position from midline, two positions from midline). In the post-hoc tests we report the p. Hochberg.

Results

Time window: 350-500

- Main effect: Semantic relatedness ($F(1,22)=27.77$, $p<.001$). Main effects averages are shown in the next Table:

Semantic related	-2.11
Semantic unrelated	-3.91

- Double Interaction: Semantic relatedness and DML ($F(1,22)=29.93$, $p<.001$) and Semantic relatedness and AP ($F(4,88)=7.06$, $p<.01$; $\varepsilon=.37$), post hoc test showed for Semantic relatedness a significant effect ($p<.01$) in all levels of Anterior Posterior.

Time window: 500-650

- Main effects: Semantic relatedness ($F(1,22)=67.07$, $p<.001$) and Sensory-Motor relatedness ($F(1,22)=10.57$, $p<.01$). Main effects averages are presented in the next Table:

Semantic related	0.77
Semantic unrelated	-1.91
Sensory-Motor related	-0.12
Sensory-Motor unrelated	-1.02

- Double Interactions: Semantic relatedness and DML ($F(1,22)=48.55$, $p<.001$) Sensory-Motor relatedness and DML ($F(1,22)=4.99$, $p<.05$). Post-hoc testing revealed a significant effect for as well Semantic relatedness ($p<.001$) as well as Sensory-Motor relatedness ($p<.05$). And finally there was a double interaction of Semantic relatedness with AP ($F(4,88)=9.15$, $p<.01$; $\varepsilon=.38$). Post hoc test showed for the last interaction a significant effect of Semantic relatedness ($p<.001$) in all levels of AP.
- Triple interaction: Semantic relatedness with DML and AP ($F(4,88)=3.24$, $p<.05$; $\varepsilon=0.73$). Post hoc test showed for this interaction a significant effect of Semantic relatedness ($p<.001$) in all levels of AP and DML, thus providing evidence for a significantly large and widespread effect.
- Quadruple interaction: Semantic relatedness with DML, Hemisphere and AP ($F(4,88)=3.17$, $p<.05$).

Appendix E

Grand averages for all the individual electrode sites for Experiment 1 and Experiment 2.

1) Experiment 1: 4 conditions (20 electrodes)

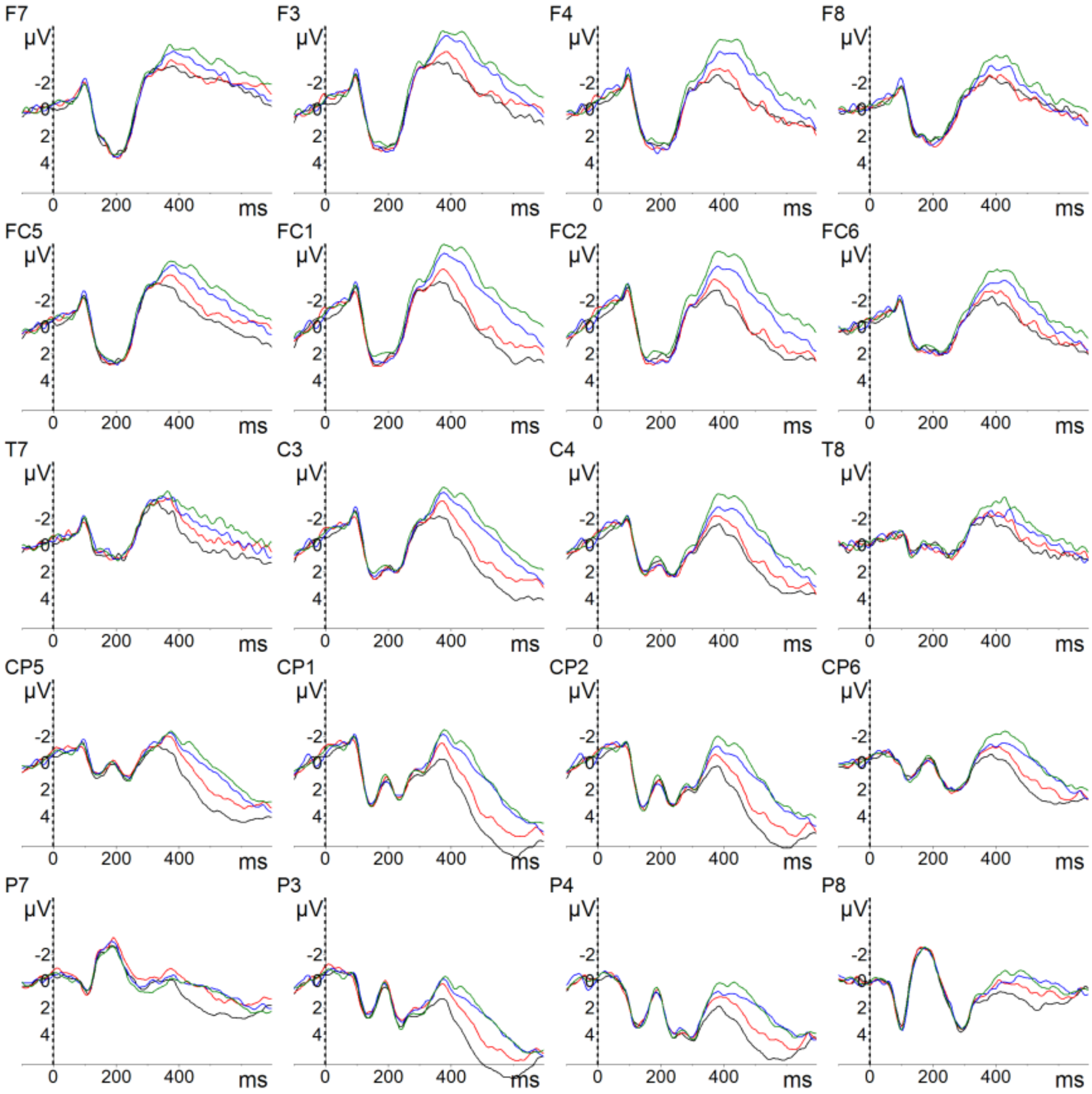

SENSORY-MOTOR RELATED-SEMANTIC RELATED

SENSORY-MOTOR RELATED-SEMANTIC UNRELATED

SENSORY-MOTOR UNRELATED-SEMANTIC RELATED

SENSORY-MOTOR UNRELATED-SEMANTIC UNRELATED

2) Experiment 2: 4 conditions (20 electrodes)

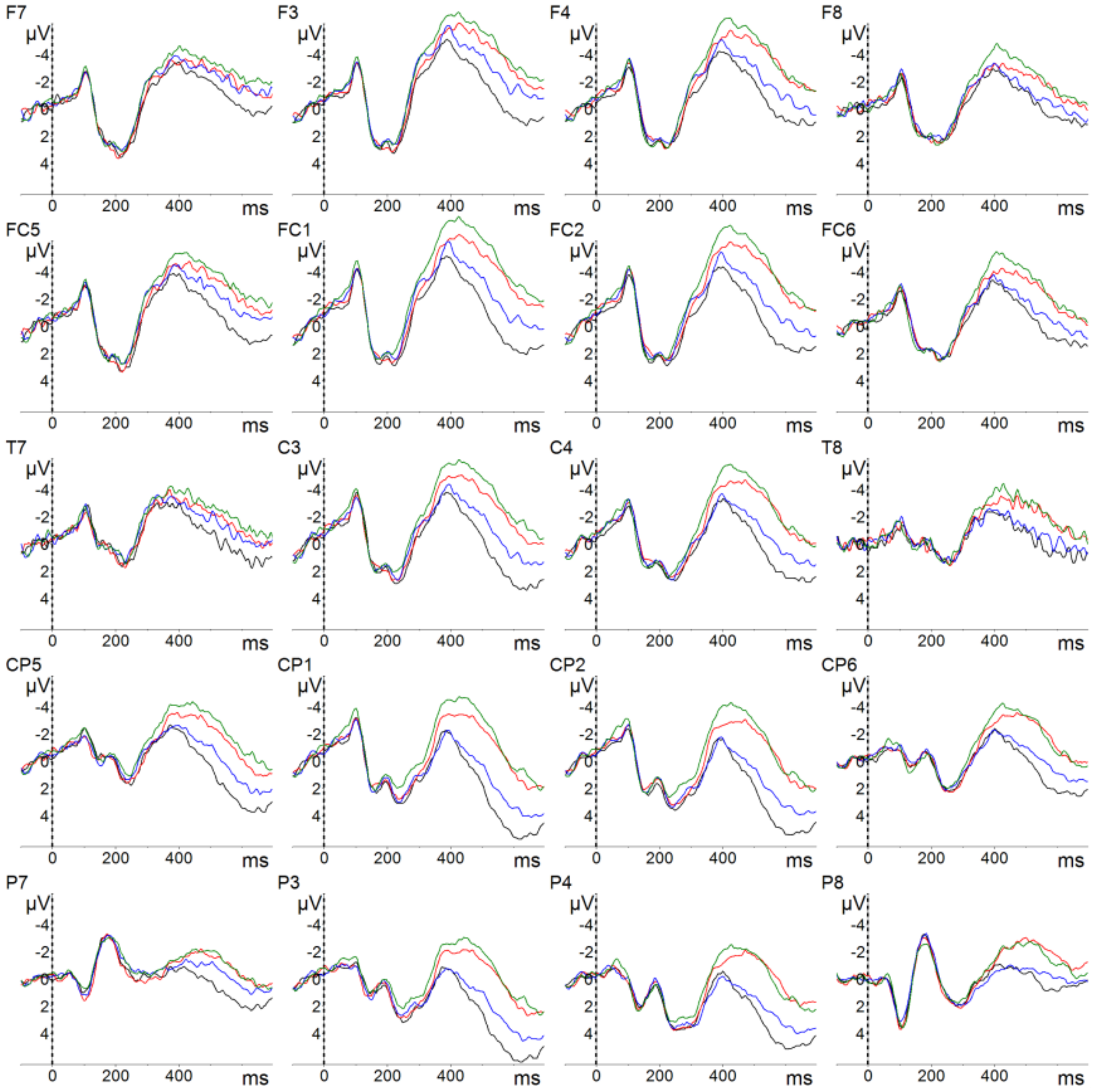

SENSORY-MOTOR RELATED-SEMANTIC RELATED

SENSORY-MOTOR RELATED-SEMANTIC UNRELATED

SENSORY-MOTOR UNRELATED-SEMANTIC RELATED

SENSORY-MOTOR UNRELATED-SEMANTIC UNRELATED

Appendix F

Experiment 2. T-test values and associated probability for pairwise analyses of the four experimental conditions at the nine interest areas in the two ERP temporal windows (significant differences are in gray background).

Experiment 2: Implicit sensory-motor relations (explicit semantic encoding)			
Contrast	Region	350 - 500 time window	500 - 650 time window
Sensory-motor Related (SMT-R)			
SEM-R vs. SEM-U	Left - Anterior	$t = 2.84$, $p = 0.01$	$t = 5.04$, $p = 0.0001$
	Left - Central	$t = 3.96$, $p = 0.0001$	$t = 7.59$, $p = 0.0001$
	Left - Posterior	$t = 4.60$, $p = 0.0001$	$t = 7.95$, $p = 0.0001$
	Medial - Anterior	$t = 3.70$, $p = 0.001$	$t = 6.27$, $p = 0.0001$
	Medial - Central	$t = 4.72$, $p = 0.001$	$t = 8.39$, $p = 0.0001$
	Medial - Posterior	$t = 4.92$, $p = 0.0001$	$t = 7.97$, $p = 0.0001$
	Right - Anterior	$t = 2.35$, $p = 0.03$	$t = 3.64$, $p = 0.001$
	Right - Central	$t = 3.86$, $p = 0.001$	$t = 5.49$, $p = 0.0001$
	Right - Posterior	$t = 4.29$, $p = 0.001$	$t = 6.97$, $p = 0.0001$
Sensory-motor Unrelated (SMT-U)			
SEM-R vs. SEM-U	Left - Anterior	$t = 2.46$, $p = 0.02$	$t = 2.56$, $p = 0.018$
	Left - Central	$t = 3.07$, $p = 0.005$	$t = 2.95$, $p = 0.007$
	Left - Posterior	$t = 3.23$, $p = 0.004$	$t = 3.43$, $p = 0.002$
	Medial - Anterior	$t = 2.82$, $p = 0.01$	$t = 2.94$, $p = 0.007$
	Medial - Central	$t = 4.69$, $p = 0.001$	$t = 3.98$, $p = 0.001$
	Medial - Posterior	$t = 3.75$, $p = 0.001$	$t = 3.47$, $p = 0.002$
	Right - Anterior	$t = 2. 75$, $p = 0.02$	$t = 2.88$, $p = 0.009$
	Right - Central	$t = 3.07$, $p = 0.005$	$t = 3.12$, $p = 0.005$
	Right - Posterior	$t = 3.25$, $p = 0.003$	$t = 3.44$, $p = 0.002$
Semantic Related (SEM-R)			
SMT-R vs. SMT-U	Left - Anterior	$t = 1.44$, $p = 0.16$	$t = 2.60$, $p = 0.016$
	Left - Central	$t = 2.23$, $p = 0.04$	$t = 3.83$, $p = 0.001$
	Left - Posterior	$t = 1.74$, $p = 0.09$	$t = 3.55$, $p = 0.002$
	Medial - Anterior	$t = 1.95$, $p = 0.06$	$t = 3.24$, $p = 0.004$
	Medial - Central	$t = 1.46$, $p = 0.16$	$t = 3.49$, $p = 0.002$
	Medial - Posterior	$t = 0.43$, $p = 0.66$	$t = 2.81$, $p = 0.01$
	Right - Anterior	$t = 0.81$, $p = 0.42$	$t = 1.69$, $p = 0.105$
	Right - Central	$t = 1.13$, $p = 0.27$	$t = 2.95$, $p = 0.007$
	Right - Posterior	$t = 0.67$, $p = 0.51$	$t = 3.62$, $p = 0.001$
Semantic Unrelated (SEM-U)			
SMT-R vs. SMT-U	Left - Anterior	$t = 0.58$, $p = 0.57$	$t = 0.43$, $p = 0.66$
	Left - Central	$t = 0.64$, $p = 0.52$	$t = 0.63$, $p = 0.53$
	Left - Posterior	$t = 0.52$, $p = 0.61$	$t = 0.43$, $p = 0.67$
	Medial - Anterior	$t = 0.74$, $p = 0.46$	$t = 0.40$, $p = 0.69$
	Medial - Central	$t = 1.14$, $p = 0.26$	$t = 0.53$, $p = 0.60$
	Medial - Posterior	$t = 0.17$, $p = 0.86$	$t = 0.73$, $p = 0.47$
	Right - Anterior	$t = 1.09$, $p = 0.28$	$t = 0.35$, $p = 0.72$
	Right - Central	$t = 0.77$, $p = 0.44$	$t = 0.25$, $p = 0.80$
	Right - Posterior	$t = 0.17$, $p = 0.86$	$t = 0.51$, $p = 0.61$

Appendix G

Figure 8. Scalp distributions of the main effects the two analyzed time windows (350-500 and 500-650 ms): Sensory-motor effect after collapsing semantic conditions (left column) and Semantic effect after collapsing sensory-motor conditions (right column), separately for Experiment 1 (top) and Experiment 2 (bottom).

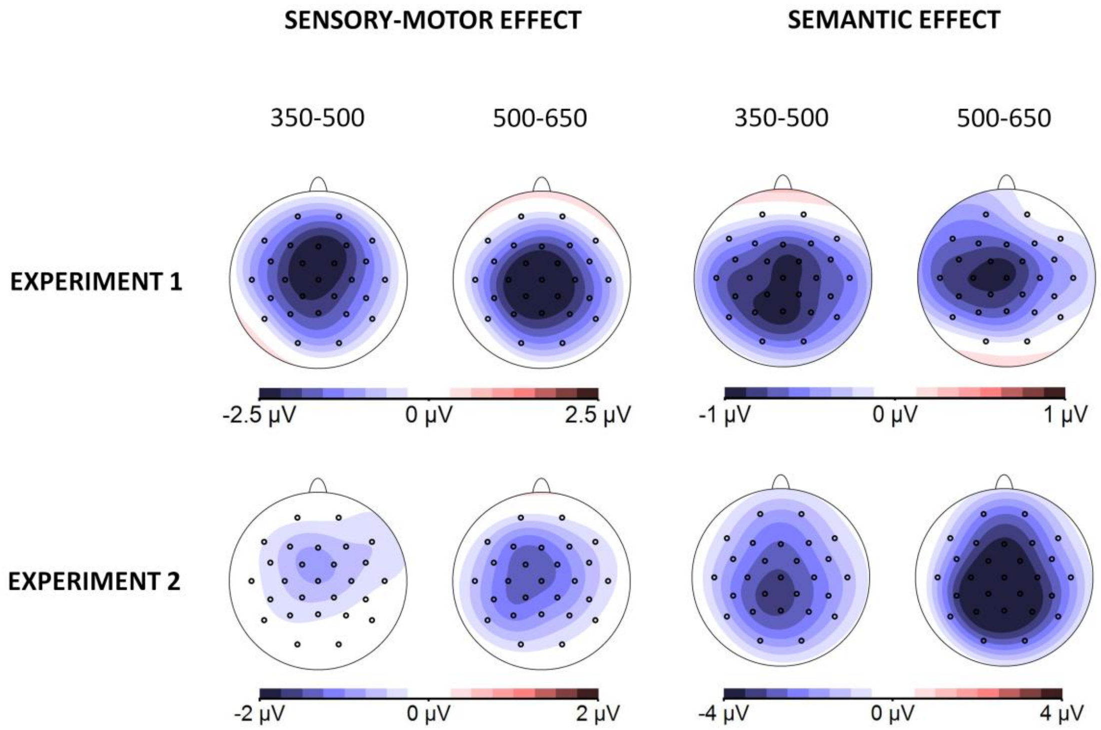

Supplement: Supplementary file 1 [file Data_Sheet_1.PDF]
